# Links between Cognitive Status and Trace Element Levels in Hair for an Environmentally Exposed Population: A Case Study in the Surroundings of the Estarreja Industrial Area

**DOI:** 10.3390/ijerph16224560

**Published:** 2019-11-18

**Authors:** Marina M. S. Cabral Pinto, Paula Marinho-Reis, Agostinho Almeida, Edgar Pinto, Orquídia Neves, Manuela Inácio, Bianca Gerardo, Sandra Freitas, Mário R. Simões, Pedro A. Dinis, Luísa Diniz, Eduardo Ferreira da Silva, Paula I. Moreira

**Affiliations:** 1 Geobiotec Research Centre, Department of Geosciences, University of Aveiro, 3810-193 Aveiro, Portugal; minacio@ua.pt (M.I.); eafsilva@ua.pt (E.F.d.S.); 2 Instituto de Ciências da Terra, University of Minho, 4710–057 Braga, Portugal; pmarinho@dct.uminho.pt; 3 Laboratory of Applied Chemistry, Department of Chemical Sciences, Faculty of Pharmacy, LAQV/REQUIMTE, Porto University, 4050-313 Porto, Portugal; aalmeida@ff.up.pt (A.A.); ecp@ess.ipp.pt (E.P.); luisa2diniz@gmail.com (L.D.); 4 CERENA, DECivil, Instituto Superior Técnico, University of Lisbon, 1049-001 Lisbon, Portugal; orquidia.neves@tecnico.ulisboa.pt; 5 Center for Research in Neuropsychology and Cognitive and Behavioral Intervention (CINEICC), University of Coimbra, 3030-548 Coimbra, Portugal; bianca.s.gerardo94@gmail.com (B.G.); sandrafreitas0209@gmail.com (S.F.); simoesmr@fpce.uc.pt (M.R.S.); 6 MARE—Marine and Environmental Sciences Centre, Department of Earth Sciences, University of Coimbra, 3030-790 Coimbra, Portugal; pdinis@dct.uc.pt; 7 CNC—Center for Neuroscience and Cell Biology, University of Coimbra and Institute of Physiology, Faculty of Medicine, University of Coimbra, 3030-548 Coimbra, Portugal; pimoreira@fmed.uc.pt

**Keywords:** exposure, trace elements, cognitive impairment, mercury

## Abstract

In the present study, trace elements (TE) levels were evaluated in scalp hair along the continuum from healthy subjects (HS) to patients suffering from subjective memory concerns (SMC), and/or mild cognitive impairment (MCI), and those with already installed dementia (DEM) in order to: (i) assess the effects of environmental and lifestyle factors on TE concentrations and (ii) evaluate the analyzed elements as possible diagnostic biomarkers for the disease. The study involved 79 mainly permanent residents, >55 years old, from the city of Estarreja (northern Portugal), a former industrial area. The health status of the participants was assessed by means of a complete socio-demographic questionnaire and through cognitive screening tests, namely the Mini-Mental State Examination (MMSE). The test scores were categorized and used in the statistical analysis. Hair samples were collected and analyzed by inductively coupled plasma-mass spectrometry (ICP-MS) ICP-MS for selected TE. Dementia appears to be associated with higher age, the female gender, lower education level, and longer residence time in the study area. In addition, most of the participants diagnosed with dementia frequently consume home-grown foodstuffs, some irrigated with contaminated well water. The calculation of the TE enrichment factors of soil samples collected in kitchen gardens/small farms in the vicinity of the Estarreja Chemical Complex (ECC) reinforces the degree of Hg soil contamination in the area, due to anthropogenic sources that can be a source for the population Hg exposure route among others. Mercury levels in hair differed significantly between the four individual groups (HS, SMC, MCI, and DEM), increasing from healthy to dementia participants. Improved diagnostic results can be obtained using hair TE signatures coupled with MMSE scores. This strategy may prove useful for predictive diagnosis in population screening for cognitive impairment.

## 1. Introduction

The aging of human populations around the world is leading to an epidemic of Alzheimer’s disease (AD), with the number of cases estimated to rise to nearly 106 million by 2050 [1]. Human AD is characterized by a progressive decline of cognitive function, with marked loss of memory and other cognitive functions, leading to a gradual loss of functionality and autonomy. It is the most frequent and fearsome form of dementia in the elderly, and its cure and prevention are among the primary challenges of modern medicine. Nowadays, it is accepted that the preclinical stage of AD can begin more than a decade before symptoms are evident, and therefore detection of preclinical stages is a critical factor to fight the disease. Mild cognitive impairment (MCI) is a prodromal stage of AD and affected individuals experience memory loss and/or other cognitive impairments greater than would be expected based on their age and level of education, but not enough to allow a diagnosis of dementia. Longitudinal studies show that MCI patients progress to overt dementia at a rate of 10–15% per year, compared with a rate of 1–2% in the control subjects [2]. This explains why MCI is now the focus of prediction studies and the target of clinical trials of new disease-modifying therapies. Research on significant memory impairment is very contradictory. On one hand, numerous studies have proven that subjective memory concerns (SMC) reflect objective cognitive impairment [3,4,5,6,7] and is associated with increased risk of MCI and dementia. For instance, a meta-analysis conducted by Mitchell and colleagues [8] shows that 6.6% of older individuals with SMC, but with no objective complaints, will convert to MCI per year, comparatively to 1% in those without SMC, and that individuals in this condition double their risk of dementia. Furthermore, there is clear evidence that SMC are a reliable predictor of conversion to dementia [9,10,11,12]. However, some other studies refute these findings by reporting a lack of value of SMC [13,14] and SMC severity [15] in predicting dementia. Additionally, a longitudinal study conducted with healthy elderly showed that SMC are not associated with significant changes in cognitive performances over time [16]. Also, a review conducted by Reid and MacLullich [17] reported inconsistency in the association between SMC and cognitive impairment.

Multiple factors have been reported as contributing to the etiology of sporadic (late-onset) AD including aging, genetics, head injury, and exposure to certain chemical compounds. Whilst the genetic component of sporadic AD risk has been increasingly recognized in recent years due to genome-wide association studies (GWAS) [18], the presence of the *APOE*ε4 allele being the strongest genetic risk factor, the role of environmental exposures and the mechanisms of their contribution to the pathogenesis of AD continues to be a subject of discussion. This is partly because of the extended time lapse between exposure and onset of the disease. However, while not all environmental contaminants and toxins have been tested in research studies in terms of their impact on the central nervous system (CNS), the risks of developing AD (and other neurodegenerative diseases, like Parkinson’s disease) in elderly persons as a result of neurologic impairments caused by environmental toxins, is established [19]. Recent studies also support close gene-environment interactions [20].

Systemic human exposure to trace elements (TE) is a common circumstance worldwide, resulting from multiple exposure pathways including inhalation of ambient particulate matter, dermal absorption of trace elements from soil and dust, and ingestion of contaminated soil/dust (through hand-to-mouth movements or clearance of particulate matter from airways by swallowing), water and foodstuff, such as agricultural crops, meat and seafood. Toxicity of TE depends upon the absorbed dose, route, and duration of exposure.

Currently, no marker exists as an AD indicator in its early stages, only mainly in its prodromal stage, and the diagnosis of the disease is still based on clinical ground. Biomarkers capable of identifying the preclinical stage of AD have the potential to open a therapeutic window in which neurons would remain responsive to treatment. Furthermore, biomarkers capable of defining the at-risk state may drive novel therapeutic strategies to finally achieve a disease-modifying status of AD. Elemental profiling is an interesting approach for understanding neurodegenerative processes, considering that compelling evidence shows that element toxic effects might play a crucial role in the onset and progression of AD [21].

The use of hair for biomonitoring purposes offers the possibility of integrating a relatively long-term exposure in a single-specimen analysis and presents important practical aspects: non-invasive sample collection and easy sample transport and storage [22]. The concentration of trace elements in hair varies greatly according to the environmental exposure of the subject. On the other hand, hair is an inert tissue and trace elements are slowly incorporated into its structure. Their concentrations do not fluctuate over a short time scale, as in blood, and thus they can be used as a long-term diagnostic tool [23]. It has been reported that several characteristics such as age, sex, ethnicity, nutrition, and geographical location might affect some element concentrations in human hair [24,25,26]. It has been shown by clinical research that the levels of specific TE in hair, especially those with higher toxic potential, may present a strong correlation with specific pathological disorders. It should be noted that aging is associated with changes in metabolism and several nutrient imbalances [27] and the maintenance of an adequate TE status, in addition to macrominerals and vitamins, is particularly important in the elderly population, for maintenance of the physiological homeostasis and prevention of several age-associated diseases [28,29]. Many studies exist in elderly populations, focused on their health status regarding a specific area of gerontological epidemiology, such as mental diseases [30] and cognitive impairment [31].

The Estarreja Chemical Complex (ECC), located near Aveiro, in the center region of Portugal, has had intense industrial activity since the early 1950s, and high environmental levels of potentially toxic elements such as arsenic (As) and mercury (Hg) have been reported at this region [32,33]. Despite the significant number of studies focusing on determining the severity and the extension of the contamination [34,35,36,37,38] only a few have tried to assess potential health effects on the resident population [39,40,41,42,43]. Hence, the area displays interesting characteristics to study the potential effect of long-term exposures to chemicals in the environment.

In the present study, trace elements levels were evaluated in scalp hair along the continuum from healthy subjects (HS) to patients suffering from SMC and/or MCI and those with already installed AD in order to: (i) assess the effects of environmental and lifestyle factors on TE concentrations in the hair of the elderly and (ii) evaluate the analyzed elements as possible diagnostic biomarkers for the disease.

## 2. Methods

### 2.1. Participants

Ethical approval for this study was obtained from the National Committee for Data Protection (nº 11726/2017). The study involved 79 participants, voluntarily recruited through convenience sampling, who met the criteria: (i) to have resided in the study area at least the last 5 previous years, in order to ensure minimum exposure time [44] and (ii) be over 55 years of age (age group of interest due to the likelihood of this population reporting subjective cognitive complaints and/or being involved in pathological aging processes, such as the dementia spectrum). 

Additionally, the following exclusion criteria were considered: (a) absence of Portuguese language skills required for cognitive testing; (b) a current or past history of neurological disease (other than SCM, MCI or dementia), traumatic brain injury, or psychiatric disorder, including depression—depression at screening was assessed with the Geriatric Depression Scale (GDS), and participants with a GDS score ≥21 [45,46,47] were considered depressed and excluded from the study; (c) previous or current alcohol or other substance abuse; (d) severe visual or auditory impairment that would negatively affect the ability to satisfactorily complete tests or understand test instructions; or (V) current or prior use of antipsychotic medication. 

The same expert neuropsychologist administered a battery of tests to all participants in a fixed order, which included the following instruments:

(1) Sociodemographic and clinical questionnaire: During a personal interview, demographic and clinical data were collected through an extensive sociodemographic questionnaire and an inventory of past habits, current clinical health status, and medical history. The following data were collected: age, marital status, weight, height, nationality, education level, the period of time working in agriculture, pesticide application methods and time of exposure, use of personal protective equipment, home-grown foodstuff consumption, irrigation water source, and drinking water source. Additionally, a full medical record was obtained during this interview, including information on 29 symptoms typically associated with toxic elements exposure or essential elements deficiency [48];
(2) The Mini-Mental State Examination (MMSE) [49,50,51] is a brief screening test for assessment of the global cognitive status. The MMSE score ranges from 0–30, with higher scores indicating better cognitive performance. In this study we considered the following categories: (i) 0–25 points: dementia; (ii) 26–28 points: mild cognitive impairment (MCI); and (iii) 29–30 points: normal cognitive functioning [51]. The MMSE is the most broadly used brief cognitive screening instrument in clinical, epidemiological, and research contexts. Despite the existence of other neuropsychological instruments with greater sensitivity in detecting cognitive decline at earlier stages (e.g., probably due to the lack of MMSE in including executive functioning assessment and the usage of rather simple tasks to assess short-term memory, working memory, attention, and concentration, language and visuospatial skills), the MMSE has been largely validated for different populations, thus representing a common reference in the communication between health professionals, including psychologists, neurologists, and psychiatrists [51];
(3) The Geriatric Depression Scale (GDS) [45,46,47] is a brief instrument to assess depressive symptoms in older adults, composed of 30 dichotomous questions that evaluate emotional and behavioral symptoms. The maximum score is 30 points, with higher scores indicating greater severity of depressive symptomatology. In this study, we considered the following categories: (i) 0–10 points: absence of depressive symptoms; (ii) 11–20 points: mild depressive symptoms; and (iii) 21–30 points: moderate to severe depressive symptoms.

### 2.2. Study Groups

The study groups were generated according to their education levels and their subjective memory complaints. The four groups were selected based on MMSE and memory scale complaints score ranges from 0–30. According to Kaup et al. [52] and O’Bryant et al. [53], the following categories were used in the statistical analysis described below: a) to lower education level [53], 0–23: dementia (DEM), 24–28: mild cognitive impairment (MCI), 29–30 (and reaching 4 on the scale of complaints): subjective memory complaints (SMC), and 29–30: healthy status (HS); b) to higher education level, 0–26: dementia, 27–28: mild cognitive impairment (MCI), 29–30 (and reaching 4 on the scale of complaints): SMC, 29–30: HS.

### 2.3. Hair Samples and Analysis

Human biomonitoring, defined as “the method for assessing human exposure to chemicals or their effects by measuring these chemicals, their metabolites or reaction products in human specimens”, involves the measurement of biomarkers in different body fluids (e.g., blood, urine, and breast milk) or tissues (e.g., nails, and hair) [22].

Hair samples (ranging from 100 to 300 mg) were collected near the scalp from 79 inhabitants of the city of Estarreja. Only hair samples presenting their natural color and from individuals residing permanently in the study area were considered for analysis.

Following a procedure reported elsewhere [54], hair samples were duly washed to completely remove exogenous contamination without significantly altering the endogenous trace element content of the sample. After washing, samples were dried in a laboratory drying oven (Raypa, Spain) at 95 °C for 40 h (the time required to achieve a constant weight). Dried samples (~0.1 mg) were mineralized through a microwave-assisted acid digestion procedure with 1 ml of concentrated HNO_3_ (>65% m/m; TraceSELECT^®^, Fluka, France) and 0.5 ml of H_2_O_2_ (≥30% v/v; TraceSELECT^®^, Fluka, Seelze, Germany) in a Milestone (Sorisole, Italy) MLS 1200 Mega high performance microwave digestion unit, equipped with an HPR 1000/10 rotor. The flowing microwave oven program (W/min) was used: 250/1, 0/2, 250/5, 400/5, and 600/5. After cooling, sample solutions were made up to 8.5 mL with ultrapure water and stored in closed propylene tubes at 4 °C until analysis.

Ultrapure water (at 25 °C the resistivity value is 18.2 MΩ cm) produced in an Aarium® pro (Sartorius, Gottingen, Germany) water purification system was used throughout the work. All lab ware was duly decontaminated by 24 h immersion in a 10% HNO3 bath and thoroughly rinsing with ultrapure water. A sample blank was prepared in each digestion run (10 samples). Average blank levels were subtracted from the samples values. For analytical quality control, the certified reference material (CRM) ERM-DB001—human hair was used, using the same acid digested procedure.

Trace element concentrations were determined through inductively coupled plasma-mass spectrometry (ICP-MS) using a Thermo Fisher Scientific (Waltham, MA, USA) iCAP™ Q instrument, equipped with standard components and accessories: a MicroMist™ nebulizer (Glass Expansion, Port Melbourne, Australia), a Peltier-cooled baffled cyclonic spray chamber, a standard quartz torch, and a two-cone interface design (nickel sample and skimmer cones). High-purity argon (99.9997%; Gasin, Leça da Palmeira, Portugal) was used as the nebulizer and plasma gas. Before each analytical series, the ICP-MS instrument was tuned for maximum sensitivity and signal stability while keeping the formation of oxides and double-charged ions to a minimum. Commercially available multi-element standard solutions (Plasma CAL, SCP Science, Baie D’Urfé, Canada) were used to prepare calibration standards. The internal standard solution was prepared from an Isostandards Material (Madrid, Spain) commercial solution. 

The limits of detection (LoD) were calculated as the concentration corresponding to three times the standard deviation of 10 replicate measurements of the blank solution (2% v/v HNO_3_).

### 2.4. Soil Samples and Analysis

Composite top layer (0–15 cm) soil samples were randomly collected from 26 kitchen gardens and/or small farms, in an area of approximately 20 km^2^ in the vicinity of the Estarreja Chemical Complex. The sample sites were selected in order to provide a representative area of the agricultural soils throughout the community that surround this industrial area. All soil samples were air-dried and sieved at 2 mm. The analysis of soils was performed by ICP-MS in ACME certified laboratory after extraction with aqua regia, and according to the laboratory standards methods and quality assurance and quality control and protocols. The accuracy and precision were checked through the analysis of certified reference materials, blank spikes and duplicates (analytical splits) of randomly selected samples. The analytical precision was better than 10%.

#### Enrichment Factor

The environmental risk was evaluated calculating the enrichment factor (EFi), proposed by Buat-Menard and Chesselet [55], and calculated as follows:

EFi = (CM/Cnor) sample/(CM/Cnor) background(1)

The enrichment factor is calculated for each element (i) and indicates the enrichment of the metal (CM: concentration of each metal) in a sample, relative to the concentration of that metal in the chosen geochemical background, after normalization (Cnor) to a conservative geogenic element, which is usually Al, Fe, Zr or Sc. The normalization points to differences caused by the sampling and by the variability of behaviors of the chemical elements in the surface geochemical processes [55]. The normalizing element used was Al, also used by Islam et al. [56,57,58]. Enrichment factors above 1.5 indicates anthropogenic influence in soil composition and it can be minor (2 < EF), moderate (2 < EF < 5), significant (5 < EF < 20), very strong (20 < EF < 40), and extreme (EF > 40) [59].

### 2.5. Statistical Techniques

Variables were examined for outliers and normal distribution by means of histograms, box plots, normal quantile-quantile plots, and the Shapiro–Wilk test. When normal distribution could not be accepted, variable transformations (square, square root, and logarithmic) were attempted. The base-10 logarithm of Al, Mn, Fe, and Pb levels, and the square root of Hg, Cu, and Zn concentrations helped to improve the distribution shape. Differences between groups were tested using the Kruskal–Wallis H test with post-hoc tests, with the results interpreted based on rank differences. A probability ≤ 0.05 was assumed as significant in testing the null hypothesis of no differences across the considered clinical conditions. The one-way analysis of covariance was used to determine whether there were significant differences in element hair contents between the study groups, while “statistically controlling” the effects of the confounding variables (covariates) that were believed to affect the results. The assumption of equality of variance was assessed by means of Levene’s test, which indicated heterogeneity of variances for hair Cu levels. Finally, post hoc pairwise multiple comparisons were performed using Bonferroni correction in order to detect significant differences between two specific groups.

## 3. Results and Discussion

### 3.1. The Study Population Cognitive Status

Clinical and demographic characteristics of the individuals recruited, divided by the four study groups, are reported in Table 1. The Kruskal–Wallis H test showed statistically significant differences for age (*χ^2^*(3) = 11.626, *p* = 0.009) between SMC and Dementia (DEM) subjects (*p* = 0.013), for the level of education (χ^2^(3) = 26.988, *p* < 0.0001) between HS and DEM (*p* = 0.001), as well as between SMC and DEM subjects (*p* < 0.001), and for the amount of time living in the city (*χ^2^*(3) = 12.662, *p* = 0.005) between HS and DEM subjects (*p* = 0.012). Table 1 shows that in this study, dementia appears to be associated with higher age, female gender, lower education level, and longer residence time in the study area, i.e., in the surrounding industrial zone. Several authors [51,60,61,62,63,64] have shown that sociodemographic variables have an important effect on cognitive-screening test performance, predominantly age and education level. Old age has been found to significantly increase the probability of obtaining lower scores, whereas the worst performance has been found among those with lower education levels and ceiling effects have been observed among highly educated individuals. The magnitude of the effect of education level is so strong that education is invariably considered a criterion for the establishment of normative data for the MMSE [65,66,67]. Some studies further suggest that high diet quality in terms of the consumption of vitamins, minerals, and trace elements can be expected when education levels are high [68,69]. However, the relationship between education and diet quality seems to be significant for deficient intake only. In our study, none of the participants were reported to have TE deficiency. 

Previous studies regarding the effect of gender have proven to be more controversial; only a few have shown a significant association between this variable and cognitive-screening test performance [70,71,72]. However, a recent study report that female *APOE* ε4 carriers have faster rates of memory decline than their male counterparts among MCI individuals [73] corroborating the observations of Iwata and colleagues [74].

In Table 1 it is also noticeable that participants whose professional activity is associated with agriculture and fisheries seem the most vulnerable to dementia. In addition, most of the participants diagnosed with dementia frequently consumed local home-grown foodstuffs. Cabral Pinto et al. [41,43,44] reported a link between cognitive status and both agricultural activity and the consumption of crops cultivated in soils irrigated with groundwater from wells in the ECC surroundings. Regarding medical history, there was no clear relation with the cognitive levels, although it was found that cardiovascular diseases were relatively common in the participants with lower cognitive level, which is in accordance with previous observations of [75].

### 3.2. TE Levels in Hair and Population Cognitive Status Relations

Biomonitoring is used as a means for assessing the impact of environmental chemical elements on living organisms [76]. Regardless of whether they are essential, nonessential, or highly toxic, human hair acts as an excretory tissue for all elements, which become incorporated into the hair matrix during its growth. In general, a target population’s health and nutrition status regarding TE can be assessed by measuring the levels in hair samples [22], i.e., the determination of TE in hair is a way of indirectly testing for the body’s overload or deficiency. 

In this study, seven TE (Al, Mn, Fe, Cu, Zn, Hg, and Pb) were measured in the hair of Estarreja residents with or at risk of AD. Among them, two TE [Zn (*χ^2^* (3) = 11.723, *p* = 0.008) and Hg (*χ^2^* (3) = 17.772, *p* < 0.001)] showed statistically significant differences in their hair concentrations between the study groups (Table 2). Pairwise comparisons showed significant differences between SMC vs. DEM subjects for Zn and between HS vs. DEM subjects for Hg. Table 2 also presents reference interval values (P5–P95) for toxic TE (Al, Hg, and Pb) estimated in accordance with International Union of Pure and Applied Chemistry recommendations [77] and reference range values for essential TE (Cu, Fe, Mn, and Zn), reported from a review of Mikulewicz et al. [78]. In general, the mean levels of the analyzed TE found in this study were well within the range reported for non-exposed people, in all the groups (four different cognitive status), except for Hg (Table 2). The mean level of Pb in hair was above the reference range in the DEM group; the mean level of Mn in hair was above the reference range in the MCI and DEM groups, and the mean levels of Zn in the DEM group were also out of reference values for many participants, but there was no statistically significant difference (*p* > 0.05). The mean level of Hg in hair was above the reference range in the SMS, MCI, and DEM groups. The TE body burden in the study groups suggests a potential long-term environmental exposure, which is evidenced by the significantly higher (*p* < 0.05) hair Hg content compared to non-exposed people. 

It has been demonstrated that several factors have the potential to influence TE concentrations in human hair (e.g., Hartmann and Kist [23], and references therein). Our study groups presented significant differences in age and education level (Table 1), suggesting that they are potential confounding variables. The effect of factors such as sex, age, education level, etc., on hair TE concentration has been shown by many authors (e.g., [26,79,80,81,82,83]). Therefore, an analysis of covariance was performed considering several variables such as age, gender, level of education, use of minerals-containing food supplements, time period of residence in Estarreja, home-grown foodstuff consumption, and type of drinking water as covariates. Three outliers were excluded from this covariance analysis, which is highly influenced by the presence of extreme values in the dataset. The homogeneity of regression slopes was tested and no interaction existed between the covariates and the independent variable. However, the hair Cu levels failed Levene’s test, and it was not possible to assume the equality of variance. These results could indicate that the significant differences in hair Zn levels between SMC and DEM subjects could result from one or more of the confounding factors, such as the time period of residence or the use/exposure of water from wells containing high levels of Zn, as reported in Cabral Pinto et al. [41]. Nevertheless, available data on the exposure of the Estarreja population through soil/dust, and home-grown foodstuffs consumption [44] did not support an excessive Zn exposure. Zinc is an essential element involved in many metabolic functions and is crucial for a healthy body status [84]; however, excess Zn can be harmful and cause toxicity [85]. Excessive ingestion of Zn can suppress Cu and Fe gastrointestinal absorption [84,86]. Toxicity associated with excessive exposure to Zn is not well known. Situations in which toxicity has been observed include inhalation of zinc fumes, deliberate ingestion, exposure to contaminated food and/or drinking water and epidemiological causes [85,87]. Occupational and environmental (chronic) exposure to specific levels of Zn has led it to be suggested as a possible cause of cognitive dysfunction and dementia ([88] and references therein). 

As previous reported, only Hg differed significantly between the four groups. Pairwise multiple comparisons showed that Hg was significantly higher in DEM compared to HS (Table 3). While none of the covariates significantly predicted (*p* > 0.050) the hair Hg content, MMSE was a good predictor of the dependent variables (*p* = 0.005). Hence, the results indicate that none of the tested covariates seems to influence the concentration of Hg in the hair of the elderly. This element has been identified as one of the most toxic nonradioactive materials known to man [89]. Although it is a naturally occurring element, anthropogenic Hg is now a major worldwide concern and is an international priority pollutant as it is persistent, bioaccumulative, and toxic even at very low levels to humans and aquatic/terrestrial ecosystems. Sensory disturbances (hypoesthesia), lack of coordination of voluntary movements (ataxia), impairment of hearing, concentric constriction of the visual field, and slurred speech (dysarthria) are some of the signs of Hg poisoning [90]. It was recently shown that circulatory levels of Hg are significantly higher in AD patients [91] and it is known that Hg favors misfolding and aggregation of amyloid beta (Aβ) protein, a neurotoxic protein present in AD patients brain [92], which supports the idea that Hg is a risk factor for this neurodegenerative disease.

The risk of mercury toxicity depends very much on the form of Hg (elemental, organic, and inorganic) and route of exposure. Due to the health risks of excessive Hg exposure, the FAO/WHO Joint Expert Committee on Food Additives established a “Provisional Tolerable Weekly Intake” (PTWI) of 4 μg kg^−1^ body weight per week for inorganic Hg and 1.6 μg kg^−1^ bw per week for MeHg [93,94]. The United States Environmental Protection Agency [76] presented a lower value for the intake of MeHg, setting a reference dose (RfD) of 0.1 μg kg^−1^ bw per day [95]. The PTWI and RfD correspond to a hair Hg concentration of 2.2 and 1.0 μg g^−1^, respectively [93,94,95]. WHO, through the analysis of neurotoxicological data, considered the Hg concentration of 50 μg g^−1^ in the human hair as the “no observed adverse effect level” value for MeHg [95]. During hair growth (~1 cm per month), circulating Hg passes from the bloodstream to the hair follicle and is incorporated into the hair shaft, where it becomes stable and is carried along its length as the hair grows, providing an accumulation pattern and the history of exposure [96,97,98,99]. Mercury concentration in scalp hair is used to assess blood Hg concentrations during hair growth [76], and the methylmercury (MeHg) exposure level can also be estimated from hair Hg levels, since approximately 80% of hair Hg is MeHg [100].

### 3.3. Trace Elements Hair Versus Risk of AD

**Figure 1:**
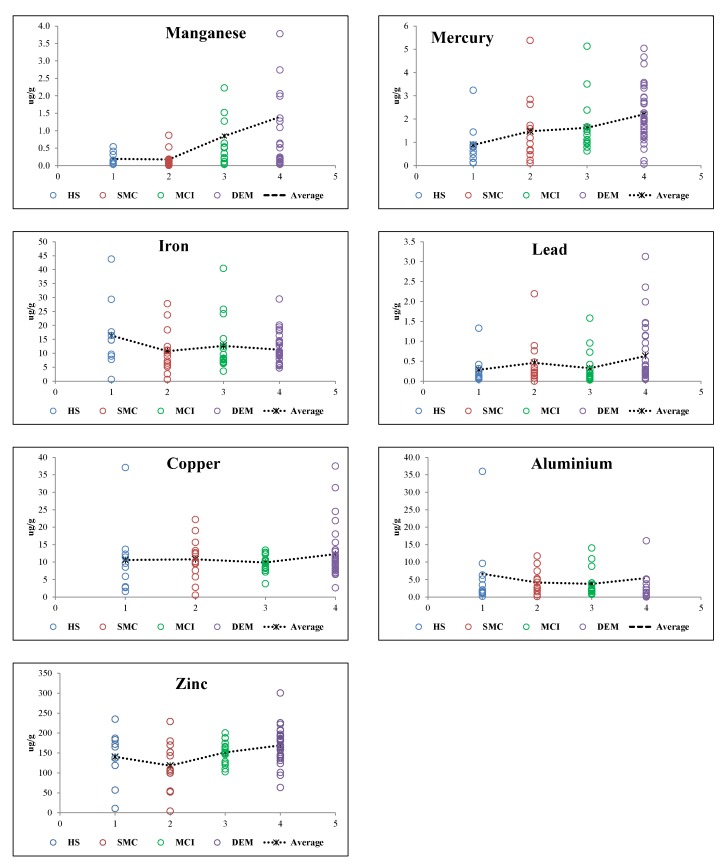
Trace elements hair levels in the different study groups.

TE are present at very low concentrations in tissues, and even small variations in these concentrations could be harmful or a sign of disease [23]. The levels of a biomarker may change over time after the onset of AD, namely from the early to the middle and the advanced stages. TE profiling is an interesting approach for understanding neurodegenerative processes, considering that compelling evidence shows that TE toxic effects might play a crucial role in the onset and progression of AD. Due to its more stable nature, the analysis of these elements in human hair is potentially more reliable than in blood [23]. Figure 1 shows a trend of an increase of Hg hair mean values between the four groups, increasing from HS to SMS, SMS to MCI (less evident), and from MCI to DEM groups. However, for the other toxic TE (Al and Pb) this signature is not followed. Lead in hair, for example shows higher mean concentrations in SMS and DEM groups. On the other hand, aluminum seems to decrease with the increase of cognitive decline groups.

Essential TE in hair (Mn, Fe, Cu, and Zn) showed a different behavior between the four cognitive statuses. Manganese and Zn in hair tended to increase from the HS to DEM group (Figure 1). Cabral Pinto et al. [42] found the highest contents of Zn and Mn in fingernails associated with the group of dements. Many studies have investigated the association between TE levels in hair and the risk of AD, but the results have also been ambiguous. Koseoglu et al. [101] found that AD patients presented significantly different concentrations of Al, Pb, Fe, Mn, Hg, and Cu in hair compared to control individuals. Vance et al. [102] compared the results of the hair analysis of 63 AD patients and 117 controls and also found that Zn levels were higher in AD patients and that no significant difference existed for the Fe concentrations, similar to our results. Koc et al. [101], in a study involving 45 patients with AD and 33 controls, found that AD patients had significantly higher hair Cu and Mn levels, but no significant differences for Fe, in accordance with our results. In contrast to our results, these authors found significantly lower Zn levels in AD patients compared to control participants; however they found no significant difference between the hair Zn levels of the two groups. Koc and co-authors [103] also found that some TE levels were changed in patients with AD. The small number of participants is a limitation of the study, which might not be enough to reach significant values in some cases.

The discovery of biomarkers that could confer high confidence to a presymptomatic AD diagnosis would be a great step forward to study the etiology of the disease, to identify the risk factors and to ultimately discover effective treatments [23]. According to the previous authors, studies such as the one presented here have the interesting potential to find reliable biomarkers in noninvasive samples for AD and MCI. However, many more studies must be conducted to allow the extraction of much more accurate information that may eventually lead to a panel of analyses that produce high-confidence AD diagnoses, especially in the early stages. The findings of our results suggest that Hg could contribute to generating a distinctive signature during the progression of dementia, and monitoring them in the elderly might help to detect preclinical stages of AD.

Plagia et al. [104] also found a variable behavior in the TE serum of subjects with or at risk of AD relatively to a control group. Paglia et al. [104] found a decreased signature for essential TE (Zn, Mn, Fe) in the serum of subjects with or at risk of AD relative to a control group. The results of a meta-analysis of Li et al. [105] provided rigorous statistical support for the association of the serum levels of TE and the risk of AD, suggesting a positive relationship between the serum Cu levels and AD risk, and a negative relationship between the serum Zn levels and AD risk. The differences between our observations and those from other authors may be simply related to the nature of the biological specimen: hair vs. serum. 

### 3.4. Metal Population Environmental Exposure

Table 4 summarizes (a) the quality of the soil samples collected in the ECC surroundings, used by the population for agricultural proposes; (b) the soil background values (BG), presented by Inácio [106], calculated by the average of Cambisoil and Podzol toplayers representative soils of the study area; (c) and the Canadian Environmental Quality Guidelines for agricultural use [107]. Manganese, iron, and aluminum have no international guidelines for any use, but their mean values in the sampled agricultural soils are impoverished relative to the mean local background values. The content of Cu, Hg, Pb, and Zn of the agricultural soil were higher than the background values, particularly Hg, Cu, and Pb (100%, 46%, and 42% of the samples). In general, the mean values of metals in soils are below Canadian limits, except for the Hg mean value which exceeded the Canadian guideline for agricultural uses. However, in some soil sampling locations, maximum Cu concentrations were also well above the Canadian guidelines for agricultural proposes. 

As previous reported, the enrichment factor (EF) for studied metals [55] has been employed for calculating differences between the metals originating from human activities and those from natural sources and it can also be used to assess and explain the contamination of metals in soils [108]. To access the degree of soil contamination through this factor, the local background values were used as it is easier to isolate anthropogenic factors from the geogenic ones, because when using generalized values, spurious enrichment due to natural local concentrations of elements may appear [109,110,111,112].

**Figure 2:**
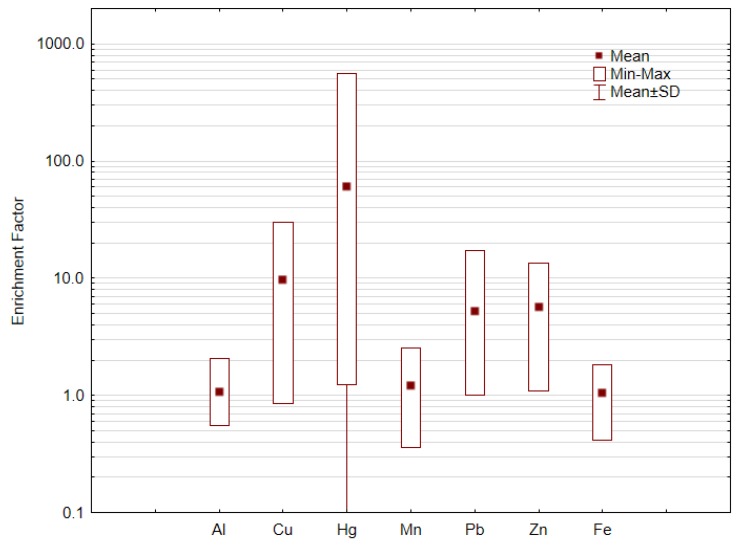
Enrichment factors for the studied elements in sampled agricultural soils from ECC surroundings.

The calculated EF values are presented in Figure 2. Considering the average EF value for each element, the enrichment decreases in the order: Hg > Cu > Zn > Pb > Mn > Fe = Al. The observed EF values indicate that 26% of the sampled agricultural soils are very strong and extremely contaminated in Hg and 65% of the samples reach significant contamination; 58%, 42%, and 27% of the sampled agricultural soils reach significant contaminant in Cu, Zn, and Pb, respectively. The soil enrichment on these elements is mainly due to the former industrial activity and agriculture practices that were greatly lowering soil quality. The degradation of soil quality by metals/metalloids pollution causes environmental risks, leads to groundwater pollution, is harmful for human health and lowers human life quality.

Biomonitoring and cognitive status relations of residents’ hair in the ECC surrounding area studied in this work highlight that Hg has features which distinguish it from remaining studied chemical elements and could be the result of long-term environmental exposure. The causes of total resident exposure to Hg may be due to the use of contaminated water for cooking and showering, inhalation, ingestion, and dermal contact of soil/dust, consumption of Hg-contaminated home-grown foodstuffs, and even fish.

The assessment of health risks for ECC surrounding residents done by Cabral Pinto et al. [44] showed that the elements of greatest concern were As and Hg, regarding both carcinogenic and non-carcinogenic risk. Reis [112] detected that total Hg concentrations in well water samples ranged between 26 and 846 ng L^−1^, and all samples presented concentrations below the maximum level allowable for drinking water as defined in Portuguese law (1.0 μg L^−1^). Even at low concentrations, water from these wells that was used for irrigation may be a problem due to the Hg bioaccumulation and biomagnification capacity associated with high toxicity. The highest concentration was detected near the S. Filipe effluent (10 m away). However, Cabral Pinto et al. [43] reported a mean/max total Hg concentration of 23.6/659 μg L^−1^ (in 2006), 60/473 μg L^−1^ (in 2010) and 1.2/4 μg L^−1^ in groundwater (in 2013). In this sampling campaign, these Hg levels exceeded both the limit for Portuguese drinking water and the groundwater intervention value of the Dutch legislation [113] for soil remediation (0.3 µg L^−1^). When this groundwater was considered for ingestion and dermal contact exposure, Hg concentrations such as those reported for some locations may constitute a non-cancer health risk.

According to Cabral Pinto et al. [44], fresh cabbage leaves (*Brassica Oleracea* L.), one of the home-grown foodstuffs consumed by the residents, yielded a total Hg concentration of 0.012 mg/kg in 4% of the studied samples, which is slightly above the limit of 0.01 mg/kg for fresh vegetable consumption, proposed in 2005 by the Ministry of Health of the People’s Republic of China [114]. Reis [112] also observed a Hg concentration of 0.01 to 0.42 mg/kg in different fish captured in the Ria de Aveiro (site of effluent discharges for many years, among others, from the Estarreja chlor-soda industrial plant). These Hg concentrations did not exceed the limits defined in Commission Regulation (EC) No. 466/2001 of 8 March (0.5 mg kg ^–1^ fresh weight) or official journal of European Communities [115] recommendation (1.0 mg kg^−1^). 

## 4. Conclusions

The inter-disciplinary approach applied in this study was successful in identifying links between different datasets. The following conclusions can be drawn:

- Participants whose professional activity was associated with agriculture and fisheries were shown to be the most vulnerable to dementia.
- Participants diagnosed with dementia frequently consume home-grown foodstuffs, some of them probably irrigated with contaminated well water.
- Biomonitoring and the analysis of the cognitive status of the residents surrounding ECC suggest that Hg levels in hair differed significantly between the four cognitive groups (healthy, subjective memory complaint, mild cognitive impairment, and dementia), increasing from healthy to dementia participants.
- Mercury mean levels in soil samples were above Canadian guidelines for soil for agricultural uses and the enrichment factor calculation values highlighted that 26% of the studied soils reached the “extremely contaminated” class for Hg, with 65% of the soils reaching the “significant contamination” class.
- Improved diagnostic results can be obtained using hair TE signatures coupled with MMSE scores. This strategy may prove useful for predictive diagnosis in populations screening of cognitive impairment.

The discovery of biomarkers that could confer high confidence to presymptomatic AD diagnosis would be a great step forward to study the etiology of the disease, to identify the risk factors, and to ultimately discover effective treatments. Supplementary studies must be conducted to achieve an adequate panel of analyses capable of providing high-confidence AD diagnoses, especially in the early stages. Our research suggest that Hg could contribute to generate a distinctive signature during the progression of dementia, and the monitoring of this in the elderly might help to detect preclinical stages of AD.

## Figures and Tables

**Table 1:** Demographic, lifestyle habits, and clinical characteristics of the study groups. Healthy subjects (HS), patients suffering from subjective memory concerns (SMC) and/or mild cognitive impairment (MCI), and those with already installed dementia (DEM).

		HS	SMC	MCI	DEM
		*n* = 10	*n* = 14	*n* = 16	*n* = 39
Age (mean ± SD)		74.0 ± 9.6	73.3 ± 7.2	78.4 ± 7.8	81.7 ± 9.0
Gender (n; %)	Male	2; 20%	2; 14%	6; 37%	3; 8%
	Female	8; 80%	12; 86%	10; 63%	36; 92%
Level of Education (mean ± SD)		7.40 ± 5.10 **	4.43 ± 2.34 **	2.88 ± 1.54	1.91 ± 3.36 **
Time of residence (mean ± SD)		53.20 ± 17.76 *	58.36 ± 27.80	58.69 ± 26.64	70.82 ± 26.08 *
Profession	Housewife	2; 20%	2; 14%	4; 25%	8; 21%
	Agriculture/Fishery	2; 20%	3; 21 %	4; 25%	16; 41%
	Industry/Construction	-	5; 36%	4; 25%	5; 13%
	Commerce/Services	6; 60%	4; 29 %	4; 25%	10; 26%
Medical History (*n*, %)					
Diabetes		-	-	2; 13%	6; 15%
Dyslipidemia		1; 10%	3; 21%	-	1; 3%
Cardiovascular		3; 30%	5; 36%	8; 50%	9; 25%
Respiratory		3; 30%	1; 7%	1; 6%	2; 5%
Other		2; 20%	3; 21%	2; 13%	5; 13%
Lifestyle factors (*n*; %)					
Supplements		-	5; 36%	3; 19%	7; 18%
Homegrown food		7; 70%	11; 79%	13; 81%	26; 67%
Drinking Water	Bottled	6; 60%	11; 79%	12; 75%	22; 56%
	Bottled & tap water	2; 20%	1; 7%	-	1; 3%
	Tap water	2; 20%	1; 7%	4; 25%	11; 28%
	Well or borehole	-	1; 7%	-	-

* (*p* < 0.05); ** (*p* < 0.01); age, level of education and time of residence are expressed in years; SD: standard deviation.

**Table 2:** Trace elements levels (µg/g) in hair samples of the study participants, reported as mean ± standard deviation (SD) and (range) according to the cognitive status (HS = healthy subjects; SMC = subjective memory complaints; MCI = mild cognitive impairment; DEM = Dementia). Also present are hair reference value ranges for non-exposed people reported from Skalny et al. [77] and Mikulewicz et al. [78].

Element	HS	SMC	MCI	DEM	Kruskal-Wallis		Pairwise Comparisons ^(a)^ (*p*-Value)						Hair Reference Values *
µg/g	*n* = 10	*n* = 14	*n* = 16	*n* = 39	H(3)	*p*-Value	DEM vs. HS	DEM vs. SMC	DEM vs. MCI	HS vs. SMC	HS vs. MCI	SMC vs. MCI	
Al	6.67 ± 10.69	4.18 ± 3.36	3.80 ± 3.95	6.26 ± 8.76	1.222	0.748	N/A	N/A	N/A	N/A	N/A	N/A	2.91–11.63
	(0.23–35.96)	(0.16–11.70)	(0.78–14.02)	(0.51–49.08)									
Mn	0.19 ± 0.17	0.18 ± 0.24	0.85±1.38	1.39 ± 2.83	7.089	0.069	N/A	N/A	N/A	N/A	N/A	N/A	0.002–0.91
	(0.04–0.54)	(0.05–0.87)	(0.03–5.46)	(0.03–16.10)									
Fe	16.37 ± 12.24	10.79 ± 7.75	12.67 ± 9.65	18.47 ± 40.24	2.266	0.519	N/A	N/A	N/A	N/A	N/A	N/A	3.66–36.8
	(0.59–43.77)	(0.59–27.78)	(3.60–40.47)	(3.41–259.26)									
Cu	10.57 ± 10.24	10.77 ± 5.82	9.85 ± 2.54	17.99 ± 36.71	1.581	0.664	N/A	N/A	N/A	N/A	N/A	N/A	7.2–82.7
	(1.62–37.10)	(0.56–22.20)	(3.83–13.39)	(2.66–237.40)									
Zn	140.29 ± 65.66	118.17 ± 58.33	151.69 ± 28.64	258.10 ± 519.33	11.723	0.008 **	n.s.	0.006 **	n.s.	n.s.	n.s.	n.s.	30–327
	(10.70–234.86)	(3.99–229.00)	(103.00–200.38)	(63.25–3396.26)									
Hg	0.88 ± 0.92	1.48 ± 1.40	1.63 ± 1.18	4.43 ± 13.86	17.772	<0.001 **	0.001**	n.s.	n.s.	n.s.	n.s.	n.s.	0.17–1.19
	(0.12–3.24)	(0.11–5.38)	(0.63–5.13)	(0.06–88.46)									
Pb	0.29 ± 0.39	0.46 ± 0.56	0.33 ± 0.42	1.02 ± 2.11	8.839	0.077	N/A	N/A	N/A	N/A	N/A	N/A	0.19–1.39
	(0.048–1.33)	(0.001–2.20)	(0.03–1.58)	(0.03–12.75)									

^a^ Post hoc analysis using Bonferroni method; N/A: not applicable; n.s.: not significant; * (*p* < 0.05); ** (*p* < 0.01).

**Table 3:** Results of the analysis of covariance (ANCOVA) performed on the hair trace elements data.

Element	ANCOVA ^a^			Pairwise Comparisons ^b^ (*p*-Value)					
	F(3, 68)	*p-Value*	*partial η^2^*	DEM*vs*HS	DEM*vs*SMC	DEM*vs*MCI	HS*vs*SMC	HS*vs*MCI	SMC*vs*MCI
Al	0.597	0.620	0.03	N/A	N/A	N/A	N/A	N/A	N/A
Mn	1.13	0.344	0.05	N/A	N/A	N/A	N/A	N/A	N/A
Fe	0.982	0.407	0.05	N/A	N/A	N/A	N/A	N/A	N/A
Cu	0.455	0.715	0.22	N/A	N/A	N/A	N/A	N/A	N/A
Zn	2.477	0.07	0.11	N/A	N/A	N/A	N/A	N/A	N/A
Hg	4.411	0.007 *	0.18	0.005 *	n.s.	n.s.	n.s.	n.s.	n.s.
Pb	2.757	0.500	0.02	N/A	N/A	N/A	N/A	N/A	N/A

^a^ Covariates: age, education level, mineral-containing food supplements use, residence time in the study area, home-grown foodstuff consumption and type of drinking water. ^b^ Post hoc analysis using Bonferroni method; N/A: not applicable; n.s.: not significant; * (*p* < 0.01).

**Table 4:** Descriptive statistics for metal contents (mg kg^-1^) in Estarreja Chemical Complex (ECC) agricultural soils, soil top layer local background (BG) values from Inácio [104] and (CEQG) Canadian Environmental Quality Guidelines (mg kg^− 1^) for agricultural land use [107].

	Al	Cu	Hg	Mn	Fe	Pb	Zn
Minimum	0.31	3	0.03	36	0.26	6	16
Mean	0.60	33	**1.5**	120	0.66	33	84
Median	0.56	23	0.15	110	0.62	23	67
Maximum	1.15	103	14	255	1.15	109	199
SD	0.21	27	4	56	0.2	23	44
Cambisoil	2.14	2	0.05	251	2.39	20	55
Podzol	0.13	12	0.05	154	0.17	6	4
BG	1.14	7	0.05	203	1.28	13	30
CEQG	NA	62	0.16	NA	NA	45	290
% > BG	0	**96**	**100**	**12**	0	**84**	**96**
% > CEQG	NA	**15**	**35**	NA	NA	**23**	0

Note: In bold are the trace elements (TE) values relatively higher than the respective guidelines; SD: standard deviation.
